# Nickel-Catalyzed Stereoselective Coupling Reactions of Benzylic and Alkyl Alcohol Derivatives

**DOI:** 10.1021/acs.accounts.3c00547

**Published:** 2023-11-07

**Authors:** Claire A. Herbert, Elizabeth R. Jarvo

**Affiliations:** Department of Chemistry, University of California, Irvine, California 92697, United States

## Abstract

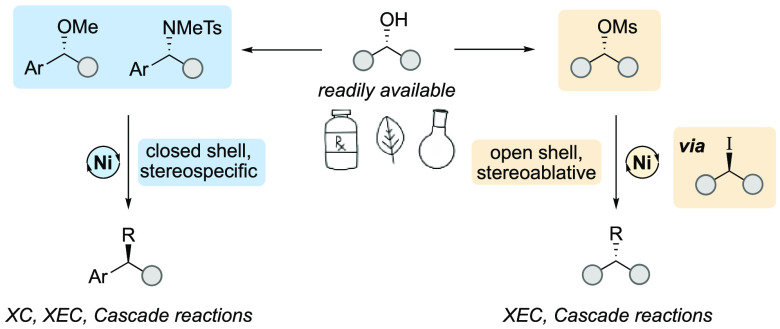

Nickel-catalyzed reactions of alkyl alcohol derivatives leverage the high prevalence of hydroxyl groups in natural products, medicinal agents, and synthetic intermediates to provide access to C(sp^3^)-rich frameworks. This Account describes our laboratory’s development of stereospecific and stereoconvergent C–C bond forming reactions employing C(sp^3^)–O and C(sp^3^)–N electrophiles. In the context of development of new transformations, we also define fundamental characteristics of the nickel catalysts.

Part I details the nickel-catalyzed cross-coupling reactions developed by our group which hinges on stereospecific formation of stable π-benzyl intermediates. Acyclic and cyclic ethers, esters, carbamates, lactones, and sulfonamides undergo Kumada-, Suzuki-, and Negishi-type coupling reactions to produce enantioenriched products with high fidelity of stereochemical information. We describe extension to include ring-opening reactions of saturated heterocycles to afford acyclic 1,3-fragments in high diastereomeric ratios. We also describe our advances in stereospecific nickel-catalyzed cross-electrophile coupling reactions. Tethered C–O and C–X electrophiles proved fruitful for construction of a variety of carbocyclic frameworks. We report an intramolecular cross-electrophile coupling of benzylic pivalates with aryl bromides for the synthesis of indanes and tetralins. We found that 4-halotetrahydropyrans and 4-halopiperidines readily undergo stereospecific ring contraction to afford substituted cyclopropanes. Mechanistic investigations are consistent with closed-shell intermediates, a Ni(0)/Ni(II) cycle, and an intramolecular S_N_2-type reaction of a key organonickel intermediate to form the cyclopropane. Building toward more complex cascade reactions, we have demonstrated that 2-alkynyl piperidines incorporate MeMgI in a dicarbofunctionalization of the alkyne to afford highly substituted vinyl cyclopropanes.

In Part II we present our development of stereoconvergent reactions of alkyl alcohol derivatives. In order to expand the utility of the intramolecular XEC reaction, we sought to employ unactivated alkyl electrophiles. Specifically, alkyl dimesylates engage in intramolecular XEC reactions to form alkyl cyclopropanes. In contrast to our previous work, these reactions proceed through open-shell intermediates and favor stereoconvergent formation of the *trans*-cyclopropane. Enantioselective aldol reactions can be employed in syntheses of 1,3-diols which furnish enantioenriched cyclopropanes in high ee. Experimental and computational evidence reveals that MeMgI mediates formation of alkyl iodides in situ. The coupling reaction initiates with halogen atom abstraction at the secondary alkyl iodide. The alkyl Ni(II) complex then proceeds through a stereospecific S_N_2-type ring closure to form cyclopropane. In an effort to increase functional group compatibility in the synthesis of cyclopropanes from alkyl dimesylates we developed a zinc-mediated reaction of 1,3-dimesylates prepared from medicinal analogues. In challenging nickel-catalyzed intramolecular cross-electrophile coupling we were also able to show that vicinal carbocycles can be prepared under similar conditions, affording vicinal cyclopentyl-cyclopropyl motifs in high yield.

In Part III we discuss our recent findings on the role of ligand identity in catalyst selectivity for stereospecific vs stereoablative mechanisms for oxidative addition. We demonstrate multivariable control of mechanism, where the choice of substrate and ligand work together to promote open- or closed-shell intermediates. In divergent reactions of 4-halotetrahydropyrans we observe distinct ligand preference for reactions at the C(sp^3^)–O center or the C(sp^3^)–Cl center. These findings are the source of continued investigations in our laboratory.

## Key References

- Chen, P.-P.; Lucas, E. L.; Greene, M. A.; Zhang, S.; Tollefson, E. J.; Erickson, L. E.; Taylor, B. L.; Jarvo, E. R.; Hong, X. A Unified Explanation for Chemoselectivity and Stereospecificity of Ni-Catalyzed Kumada and Cross-Electrophile Coupling Reactions of Benzylic Ethers: A Combined Computational and Experimental Study. *J. Am. Chem. Soc.***2019**, *141*, 5835–5855.^1^*Detailed mechanistic investigations into competing pathways for cross-coupling and cross-electrophile coupling following stereospecific oxidative addition into benzylic C(sp*^*3*^*)–O bonds.*
- Sanford, A. B.; Thane, T. A.; McGinnis, T. M.; Chen, P.-P.; Hong, X.; Jarvo, E. R. Nickel-Catalyzed Alkyl–Alkyl Cross-Electrophile Coupling Reaction of 1,3-Dimesylates for the Synthesis of Alkylcyclopropanes. *J. Am. Chem. Soc.***2020**, *142*, 5017–5023.^2^*Seminal work demonstrating nickel-catalyzed intramolecular cross-electrophile coupling of unactivated alkyl dimesylates.*
- Thane, T. A.; Jarvo, E. R. Ligand-Based Control of Nickel Catalysts: Switching Chemoselectivity from One-Electron to Two-Electron Pathways in Competing Reactions of 4-Halotetrahydropyrans. *Org. Lett.***2022**, *24*, 5003–5008.^3^*Demonstration of phosphine vs nitrogen-based ligand controlled chemoselectivity of open and closed shell intermediates in nickel-catalyzed reactions*.

## Introduction

**Figure 1:**
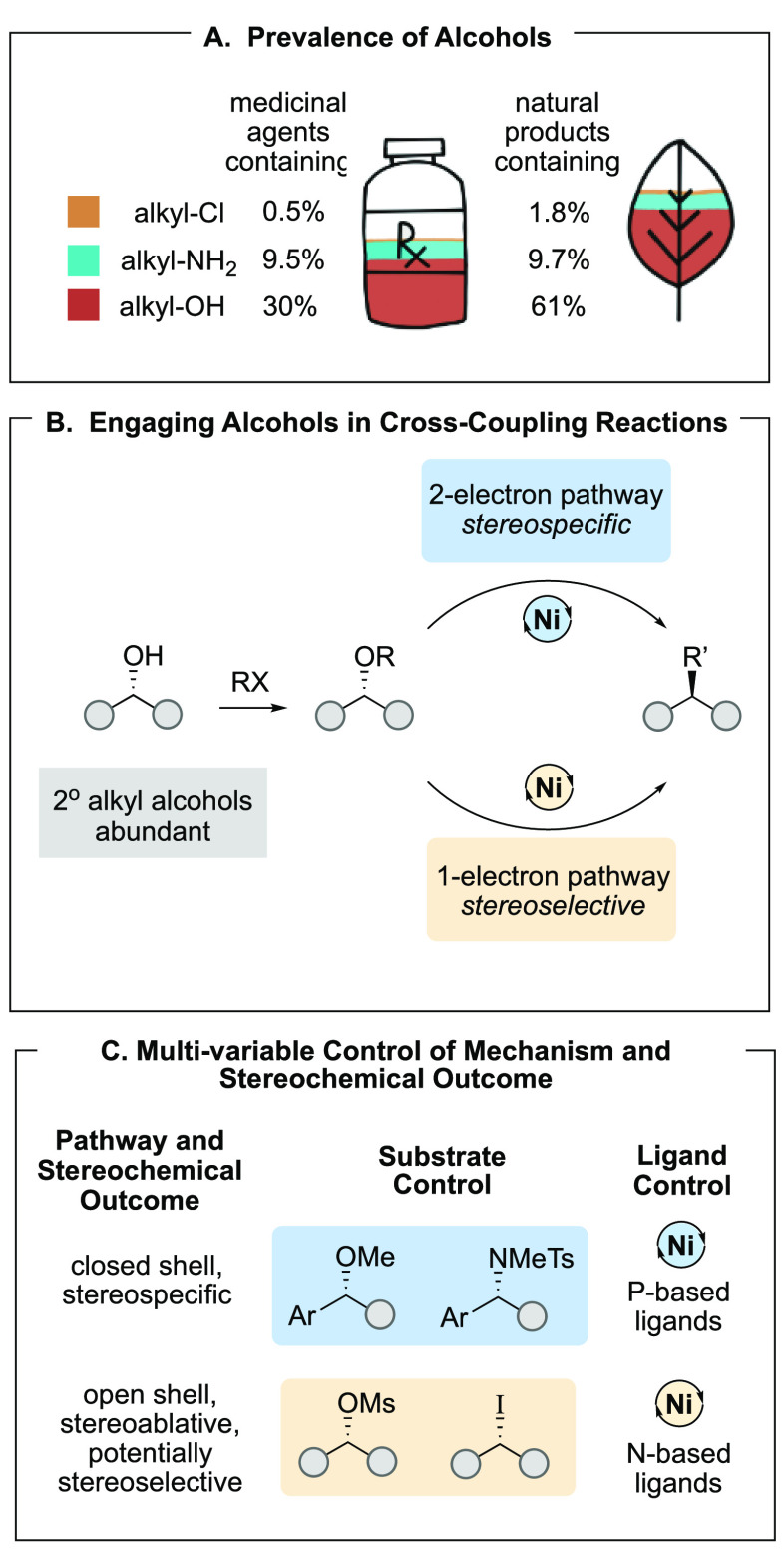
Harnessing readily available electrophiles for XC and XEC.

Introduction and control of C(sp^3^) stereogenic centers is a key challenge in synthetic and medicinal chemistry. Strategies for late-stage functionalization will have the highest impact if they engage common functional groups.^4^ We envisioned that transforming the C–O bonds of alkyl alcohol derivatives to C–C bonds, via coupling reactions, would be potentially impactful due to the natural abundance of alkyl alcohols as well as access through the versatile methods available for their synthesis (Figure 1A). In an analysis of reported natural products, alkyl alcohols were determined to have the highest frequency of all functional groups, found in over 60% of natural products.^5,6^ They also appear frequently in medicinal agents.^7^ For example, alkyl alcohols are present in 60 of the 200 highest-selling small-molecule pharmaceuticals.^6^ Furthermore, there are many stereoselective reactions for preparation of secondary alcohols as single enantiomers and diastereomers. This wide availability of complex alkyl alcohols makes them attractive as potential alkyl coupling partners in cross-coupling and cross-electrophile coupling reactions.

One obvious challenge of engaging alkyl alcohols as electrophilic coupling partners is the activation of the strong C(sp^3^)–O bond for oxidative addition. Our group is focused on developing nickel-catalyzed reactions with suitable electrophiles that can be accessed through simple synthetic manipulations to weaken the C–O bond. To this end, we have developed nickel-catalyzed transformations of ethers, esters, carbamates, lactones, and sulfonates to C(sp^3^)–C(sp^2^) and C(sp^3^)–C(sp^3^) coupled products, through traditional cross-coupling (XC), cross-electrophile coupling (XEC), and cascade reactions. In contrast to palladium catalysts more commonly employed in aryl coupling reactions, nickel catalysts are generally more reactive toward sluggish oxidative addition and less prone to deleterious β-hydride elimination.^8^ Indeed, for the transformations described here, nickel catalysts are successful while palladium catalysts often provide primarily recovered starting material or undesired byproducts.

The reactions that we have developed include both stereospecific and stereoablative coupling reactions, reflecting the diverse range of elementary steps available with nickel catalysts (Figure 1B).^9^ In the stereospecific pathway, typically observed with C(sp^3^)–O electrophiles, a two-electron S_N_2-like oxidative addition initiates the reaction. With numerous methods for the synthesis of enantioenriched alcohols, a stereospecific oxidative addition is highly advantageous since the stereochemical information on the starting materials is preserved, and cross-coupled products are produced with high enantiospecificity. Alternatively, reactions may proceed via a stereoablative pathway. Stereoablative reactions are typically observed with C(sp^3^)–X (X = I, Br, Cl) electrophiles. Oxidative addition proceeds through halogen atom abstraction (XAT) to generate an alkyl radical which can subsequently recombine with nickel to form an organonickel intermediate. This pathway allows for stereoconvergent reactions, such as diasteroselective reactions with starting materials containing pendant stereogenic centers and enantioselective reactions in the presence of a chiral ligand. Our group has developed both stereospecific and stereoconvergent reactions. In the stereospecific reactions of benzylic ethers, esters, and carbamates, we observe either net inversion or retention at the C(sp^3^) center. In reactions of alkyl mesylates, we observe rapid conversion to the alkyl iodides in situ and stereoablative reaction of the C(sp^3^) center in the presence of nickel catalysts.

Given the potential for mechanisms with very different selectivity profiles, a challenge to design selective nickel-catalyzed alkyl coupling reactions and define the key features guiding selectivity is presented (Figure 1C). Factors which favor one pathway for oxidative addition over the other remain elusive because little experimental evidence directly compares reactions that proceed through alternative pathways. However, understanding the reaction features which control these pathways would be useful for reaction design and analysis of nickel-catalyzed XC and XEC reactions. Identity of the leaving group (OR or NTsR vs I, Br, or Cl) is a critical factor in determining whether the reaction progresses through a stereospecific or stereoablative oxidative addition: ethers, esters, and sulfonamides favor two-electron oxidative addition, and halides favor one-electron halogen atom abstraction. While the electrophile plays an important role in pathway differentiation, the role of the ligand is also crucial. A typical ligand screen for nickel-catalyzed reactions can include a wide variety of phosphines, imines, amines, and N-heterocyclic carbenes. However, detailed analysis of ligand effects is typically restricted to a subset of closely related ligands. A broader understanding of the ligand influence in shunting nickel-catalyzed reactions toward open- or closed-shell intermediates is still under investigation.

In this Account, we present the developments our group has made in stereospecific and stereoselective reactions using nickel catalysts. Our work demonstrates stereoselective couplings of C(sp^3^)–C(sp^3^) and C(sp^3^)–C(sp^2^) centers in the context of cross-coupling, cross-electrophile coupling, and cascade reactions. We also discuss our progress toward defining catalyst features that control the mechanism of oxidative addition.

### Part I: Stereospecific Reactions of Alcohol Derivatives

**Figure 2:**
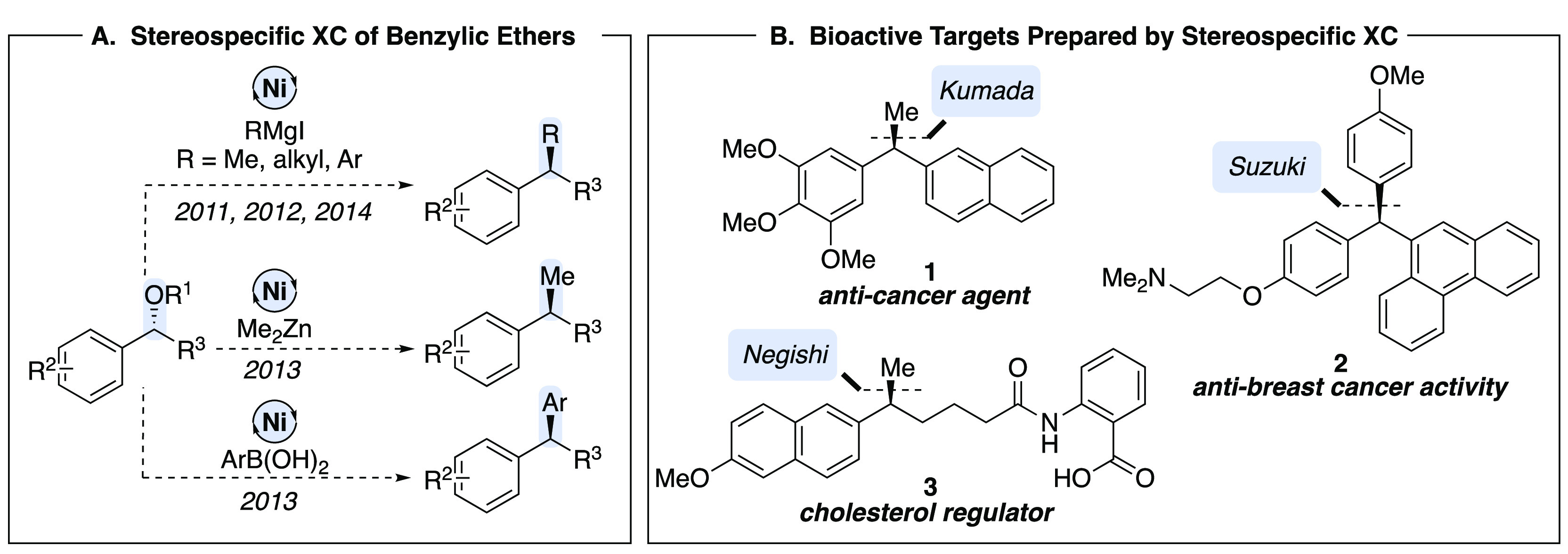
Nickel-catalyzed stereospecific XC of benzylic ethers.

To develop strategies for synthesis of C(sp^3^) stereogenic centers from alcohol derivatives, we chose to first examine benzylic ethers as cross-coupling (XC) partners. Compared to unfunctionalized alkyl alcohols, benzylic carbinols have the advantage that oxidative addition is accelerated by ligation of the arene moiety to generate a π-benzylnickel complex.^10^ We established stereospecific Kumada-, Negishi-, and Suzuki-type coupling reactions (Figure 2A). These results are consistent with two-electron concerted oxidative addition, with no open-shell intermediates. Hand in hand with reaction development, we challenged each new reaction in syntheses of compounds bearing medicinal chemistry motifs, including diaryl alkanes and triarylmethanes (Figure 2B).

**Scheme 1:**
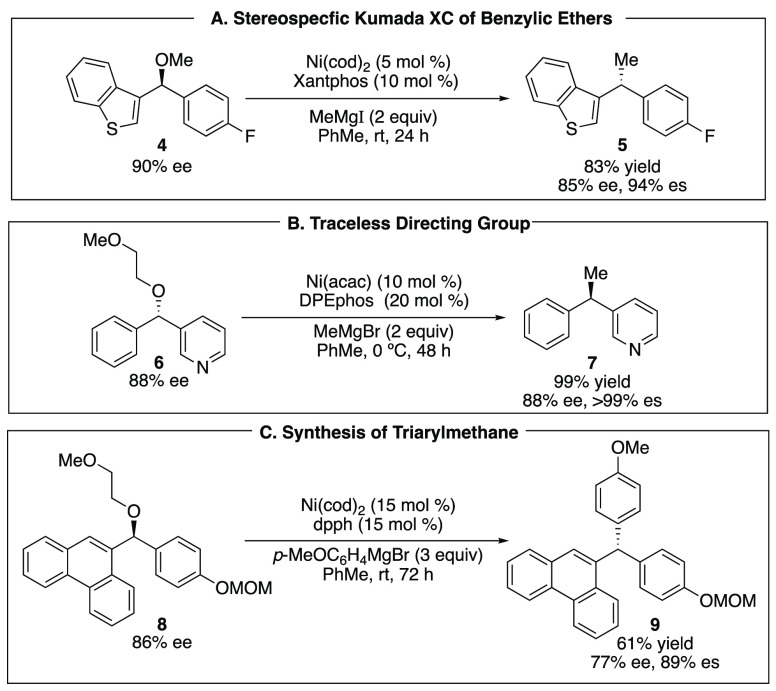
Stereospecific Kumada Coupling Reactions of Benzylic Ethers

We first harnessed the C–O bond of benzylic ethers in stereospecific Kumada-type coupling reactions with MeMgI (Scheme 1A).^11^ In the presence of Ni(cod)_2_ and bidentate phosphine ligands, enantioenriched benzylic ethers are cleanly converted to enantioenriched products with inversion at the benzylic stereogenic center. The reaction was optimized to suppress the β-hydride elimination pathway which produced styrene byproducts that were found to inhibit the nickel catalyst. To demonstrate the synthetic utility of the transformation, it was applied in the synthesis of bioactive diarylmethanes. One limitation was the requirement for activation of the C–O bond by inclusion of an extended aromatic system or heterocycle, reflecting the ability of the arene to coordinate to the low-valent nickel intermediates. Employing substrates with simple benzylic ethers resulted in recovery of starting material, likely due to challenging oxidative addition. This hurdle was overcome by development of a traceless directing group to expand the scope of the reaction to include simple arenes (Scheme 1B).^12^ This strategy also enabled access to enantioenriched triarylmethane scaffolds such as **9**, demonstrated as part of the synthesis of anti-breast-cancer agent **2** (Scheme 1C).^13^ Notably, the Watson group has also shown stereospecific activation of benzylic and allylic pivalates in Suzuki-type coupling reactions.^14^

**Scheme 2:**
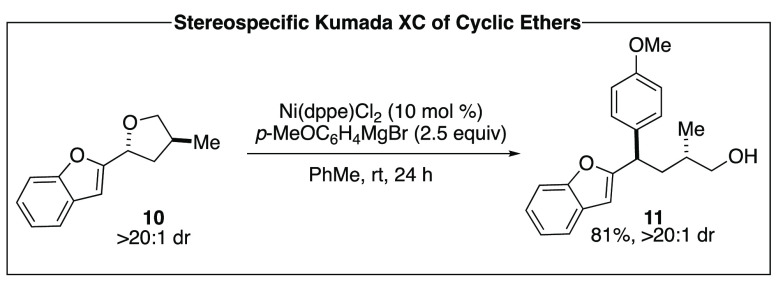
Stereospecific Kumada Coupling Reaction of Cyclic Ethers

Stereospecific reactions of cyclic ethers provide further support for robust catalyst control of configuration in nickel-catalyzed activation of C–O bonds, as well as synthetic access to stereochemically rich acyclic fragments. Tetrahydropyran and tetrahydrofuran derivatives are readily available from the corresponding aldehydes and can be synthesized as single diastereomers. These scaffolds undergo ring-opening XC to engage the benzylic C–O bond in the presence of nickel catalysts to afford acyclic coupled products in high dr (Scheme 2).^15^ Reactions proceed cleanly with inversion at the reactive center, with no catalyst match or mismatch with respect to additional stereogenic centers.

**Scheme 3:**
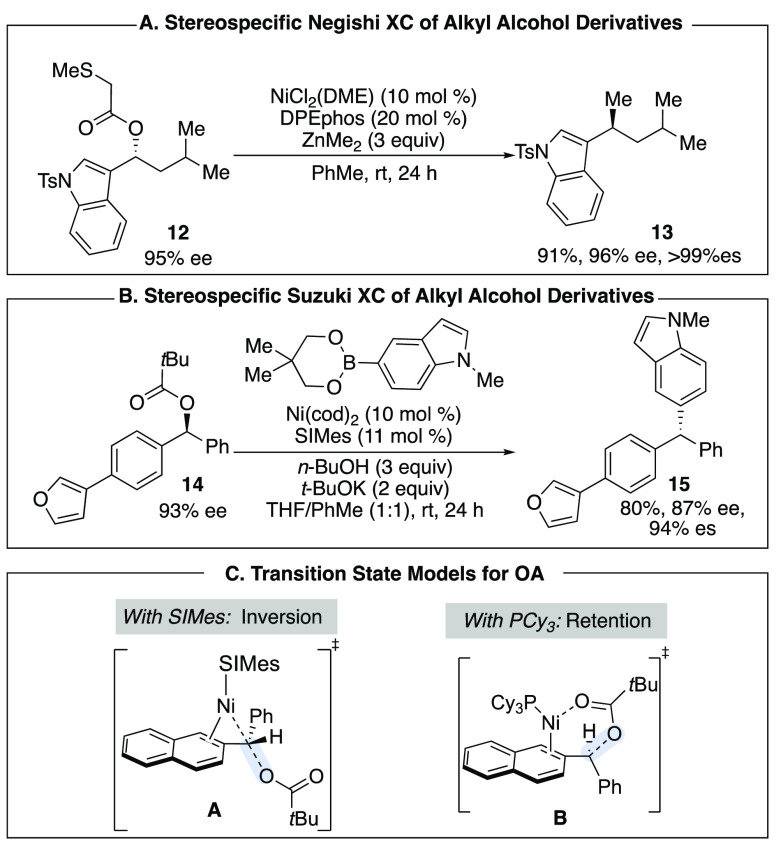
Stereospecific XC Reactions of Alkyl Alcohol Derivatives

Our laboratory has developed coupling reactions of C–O electrophiles with transmetalating agents such as boronic esters and organozinc reagents that have broad functional group tolerance. As with the Kumada-type coupling reaction to synthesize triaryl methanes, a traceless directing group was employed to activate the C–O bond toward oxidative addition for the Negishi-type coupling (Scheme 3A). In this work, the 2-methoxyethyl ether directing group employed for the Kumada-type reaction was ineffective, but the 2-(methylthio)ester afforded the desired Negishi-type coupled product in high yield and enantiospecificity.^16^ A stereospecific Suzuki coupling of benzylic carbamates and benzylic pivalates with aryl boronic esters was also developed (Scheme 3B). Interestingly, in this reaction the choice of ligand dictates whether the reaction occurs with retention or inversion at the benzylic center.^17^ Computational studies performed in collaboration with the Houk and Hong laboratories demonstrated that when SIMes ligand was employed, oxidative addition of the nickel catalyst proceeds via backside attack on the pivalate leaving group leading to net inversion in the Suzuki product (Scheme 2C, transition state **A**). However, when PCy_3_ ligand is employed, coordination of the pivalate leaving group to the nickel catalyst directs oxidative addition via a cyclic transition state that occurs with retention (transition state **B**).^18^

**Scheme 4:**
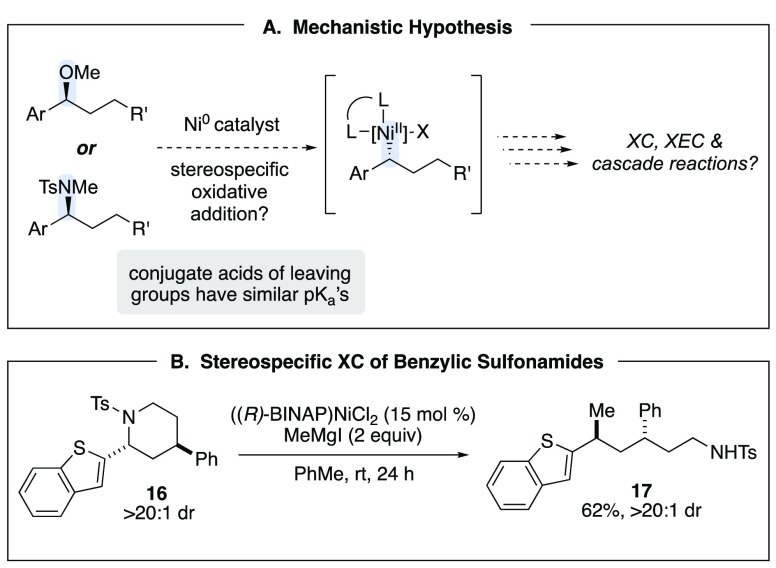
Leaving Group Stability as a Predictor for Stereospecific OA

As we learned more about the mechanisms of these reactions, we determined that oxidative addition is frequently the rate-determining step of the catalytic cycle. In the case of Kumada-type coupling reactions of benzylic ethers, oxidative addition is accelerated by coordination of Lewis-acidic magnesium salts.^19,20^ We were inspired by analogy to traditional S_N_2 and S_N_2′ reactions and sought to determine whether the guiding principles that helped design substitution reactions could also be instructive in the context of cross-coupling. In particular, we wanted to probe whether leaving groups that have conjugate acids with similar p*K*_a_’s would behave similarly in a coupling reaction, cementing the importance of the S_N_2-like oxidative addition. Sulfonamides have similar p*K*_a_’s to alcohols, and therefore we hypothesized that they would react similarly to ethers (Scheme 4A). Indeed, under the same reaction conditions employed for ethers, sulfonamides and sulfonyl piperidines such as **16** underwent ring-opening Kumada-type coupling with clean inversion at the benzylic center (Scheme 4B).^21,22^

**Scheme 5:**
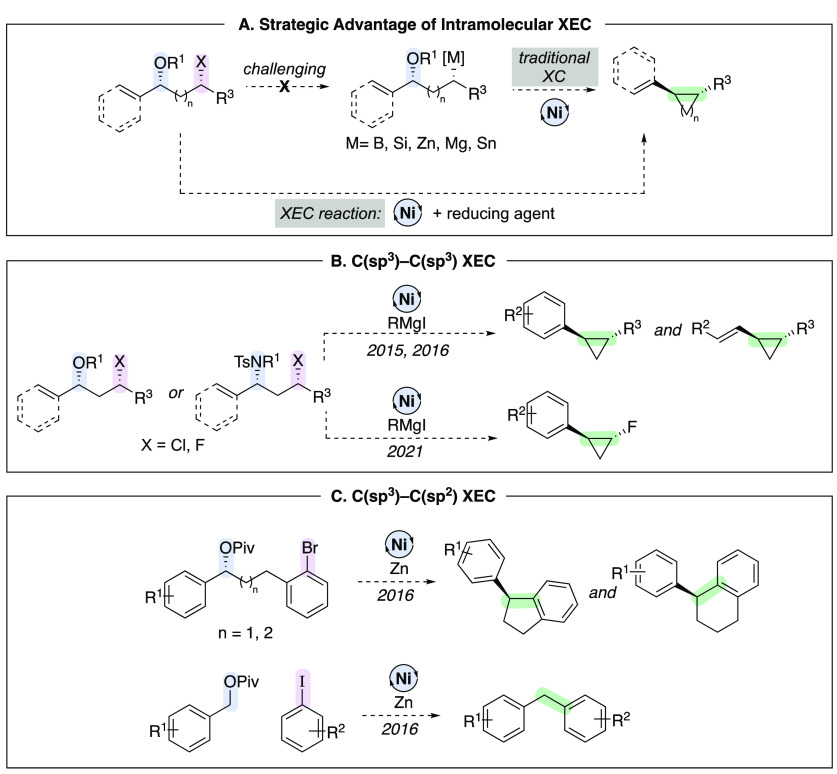
XEC as a Strategy To Overcome of Challenges of Intramolecular XC

We hypothesized that a broad range of multistep reactions could be initiated by stereospecific oxidative addition. One such application was in cross-electrophile coupling (XEC) reactions employing benzylic ethers, esters, and sulfonamides. Our early efforts focused on intramolecular reactions, where a benzylic electrophile was tethered to a second electrophile, for ring-forming and annulation reactions (Scheme 5A). Such transformations demonstrate the potential benefit of XEC over XC, since XEC eliminates the challenge of transforming one electrophile to an organometallic reagent while tethered to a second electrophile. Despite this, intramolecular variants of XEC reactions have and continue to be significantly underrepresented as compared to intermolecular XEC.^23^ We developed a series of stereospecific C(sp^3^)–C(sp^3^) coupling reactions that generate cyclopropanes (Scheme 5B) and C(sp^3^)–C(sp^2^) coupling reactions that generate indanes and tetralins (Scheme 5C). Reactions were also expanded to include intermolecular arylation of benzylic esters.

**Figure 3:**
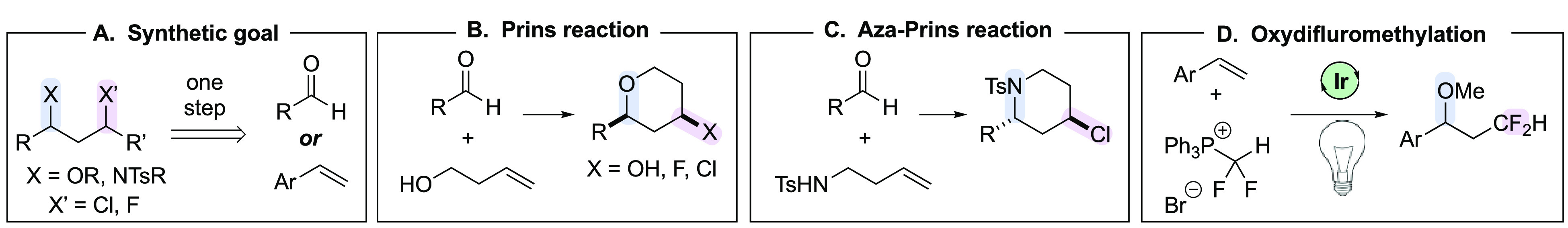
Key design principle: Rapid access to starting materials from commercially available aldehydes and olefins.

One challenge with intramolecular reactions was ensuring that starting material syntheses were practical, such that the XEC strategy could be competitive with alternative approaches toward similar carbocycles. We sought to employ starting materials that were readily available, ideally in a single step from commercial aldehydes or olefins (Figure 3A). For cyclopropane synthesis, we required synthetic access to 3-haloethers and sulfonamides, which was achieved by Prins, aza-Prins, and photochemical reactions (Figure 3B–D).

**Scheme 6:**
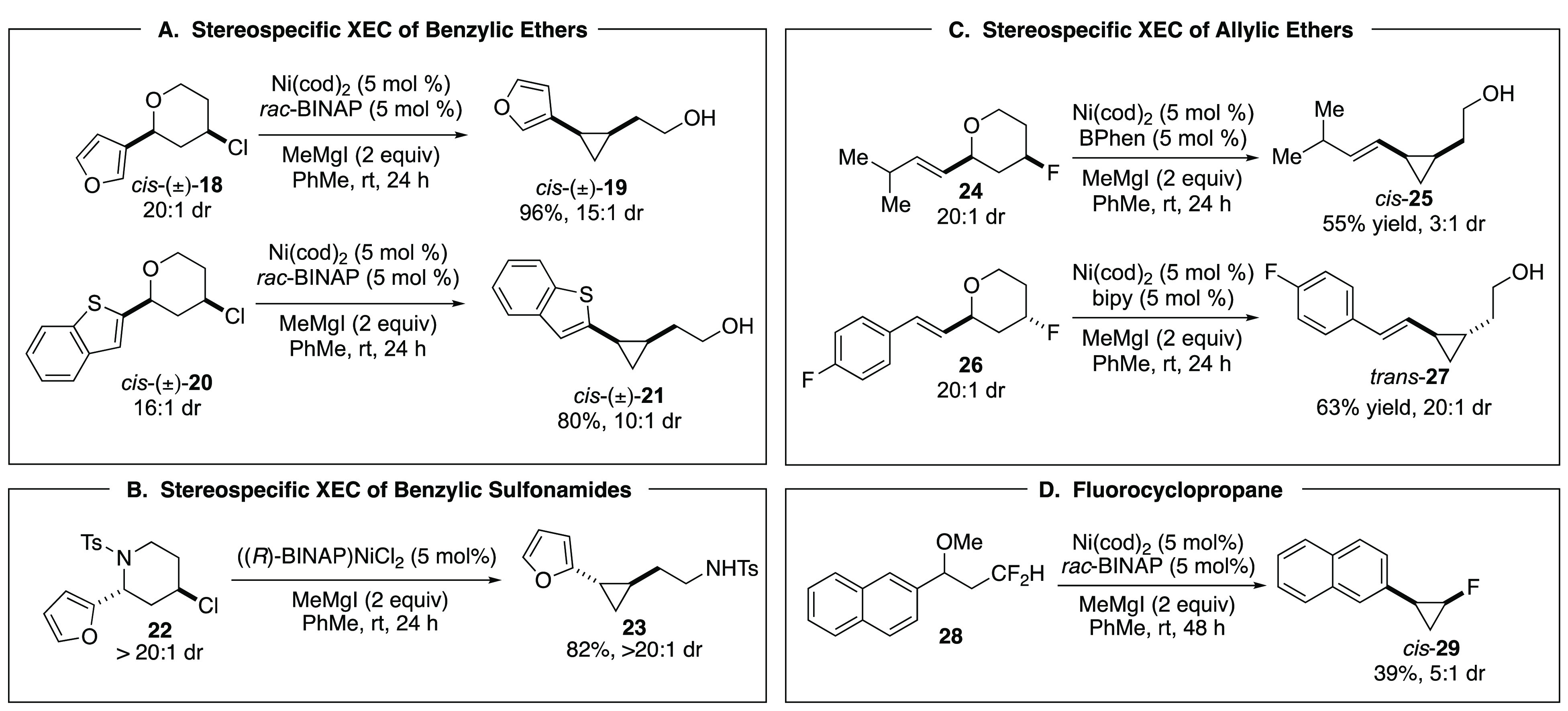
Representative XEC Reactions of Benzylic and Allylic Ethers and Benzylic Sulfonamides

Representative XEC reactions are shown in Scheme 6. Benzylic ethers and sulfonamides underwent smooth transformation in ring-contraction reactions of 4-chlorotetrahydropyrans and piperidines to afford cyclopropanes (Scheme 6A,B). Interestingly, the optimal reaction conditions were identical to the conditions for Kumada-type coupling; however, in this case the Grignard reagent served as the net reducing agent for the catalyst and was not incorporated into the reaction product. Allylic ethers also participated in intramolecular XEC reactions, including coupling with pendant alkyl fluorides (Scheme 6C). Transformations were stereospecific with respect to both alkyl electrophiles, providing robust and predictable access to either diastereomer of substituted cyclopropanes. Inspired by the success of engaging an alkyl fluoride, we challenged the reaction with an intramolecular XEC reaction of geminal difluorides (Scheme 6D). Substrates such as **28** underwent intramolecular XEC to provide highly strained fluorocyclopropanes.

**Scheme 7:**
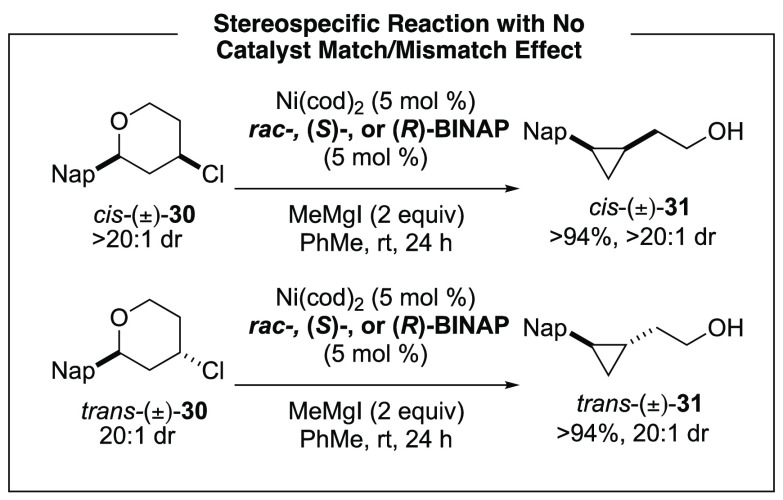
Investigation of Stereochemical Outcomes of of XEC Reaction

We were curious about the stereochemical outcomes of these transformations. In the XEC reactions where BINAP was employed as a ligand, match–mismatch experiments were conducted to probe the catalyst effects on the diastereoselectivity of the reactions. A representative test case is shown in Scheme 7. We found that the *cis*-tetrahydropyran **30** cleanly converts to *cis*-cyclopropane **31** while the other diastereomer, *trans*-tetrahydropyran **30**, affords *trans*-cyclopropane **31**, irrespective of the enantiomer of ligand. Therefore, we do not observe a catalyst match–mismatch effect for this substrate, consistent with our observations across a range of other enantioenriched substrates in XC and XEC reactions.

**Scheme 8:**
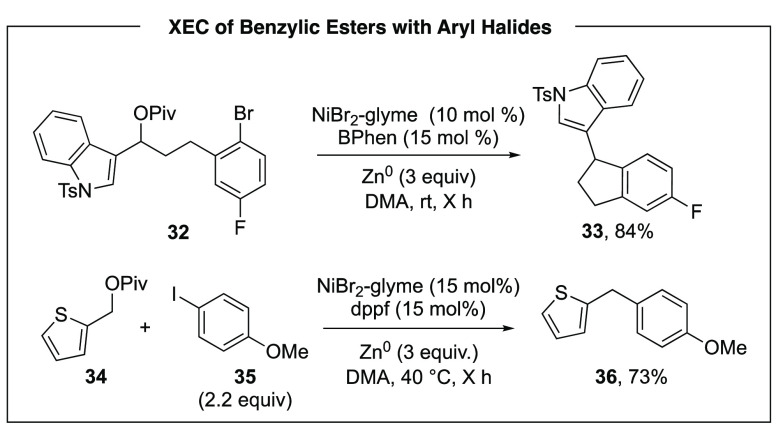
Activation of Benzylic Esters for Reductive C(sp^3^)–C(sp^2^) Coupling Reactions

We further expanded intramolecular XEC into the synthesis of 5- and 6-membered rings, by tethering benzylic esters to aryl halides (Scheme 8). This reaction provided a new synthesis of indanes and tetralins. It also translated well as a template for intermolecular C(sp^2^)–C(sp^3^) coupling reactions (Scheme 8). The mechanism is currently under investigation and is anticipated to be significantly different than C(sp^3^)–C(sp^3^) couplings, due to the sp^2^-hybridization of one of the electrophilic carbons.

**Scheme 9:**
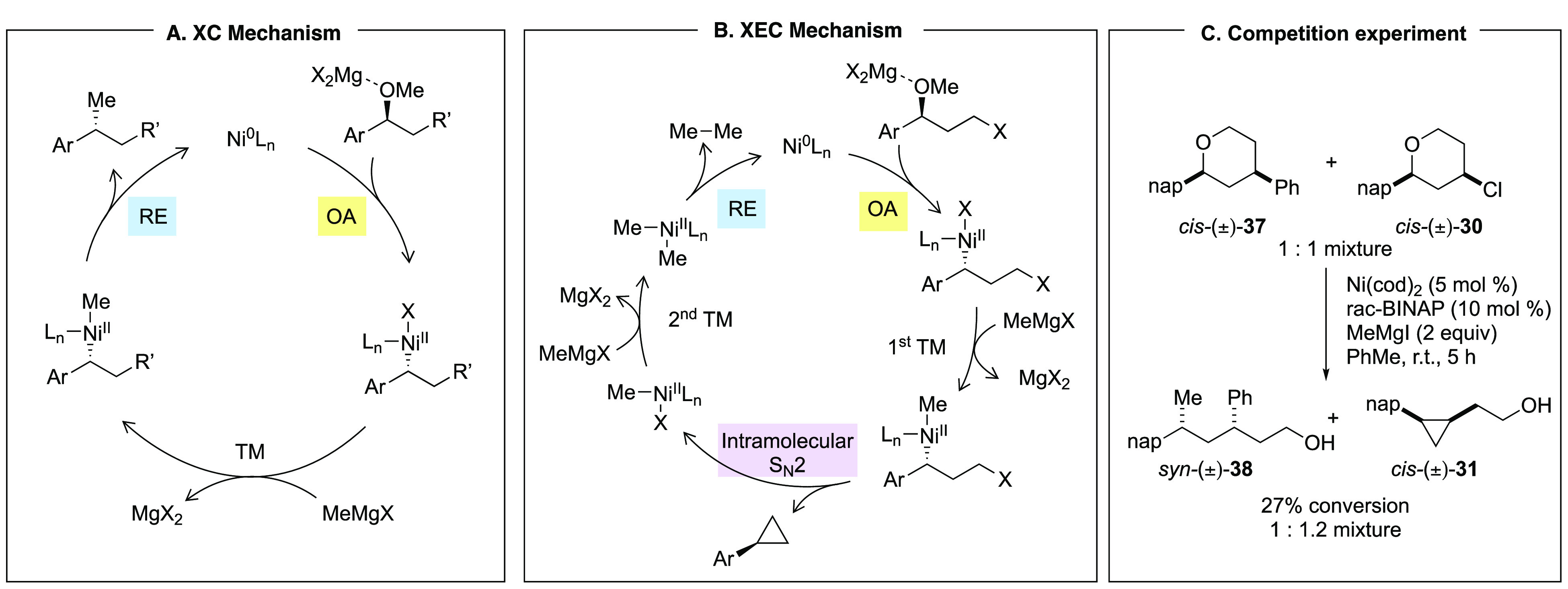
Comparison of Mechanisms for Kumada-Type XC and XEC Reactions Using Grignard Reagent

As part of reaction development, we interrogated the mechanisms of each transformation, with a particular focus on C(sp^3^)–C(sp^3^) XC and XEC reactions. Key features of important elementary steps, delineated in the context of Kumada-type reactions, translated cleanly to the mechanisms of more complex reactions including Csp^3^–Csp^3^ XEC reactions employing Grignard reagents (Scheme 9A,B).^1,24^ In particular, oxidative addition (OA) is a key step that controls the stereospecific reaction outcome. Experimental and theoretical investigations underscored that, for both the XC and XEC reactions, this elementary step initiates both catalytic cycles, is accelerated by Lewis acidic magnesium salts, proceeds with inversion at the benzylic stereogenic center, and has a barrier height of approximately 20 kcal/mol.^25^ Both mechanisms also involve a reductive elimination (RE) step that regenerates the nickel(0) catalyst from the nickel(II) intermediate, and the barrier height is often calculated to be within a few kcal/mol of the oxidative addition. A competition experiment was used to support the mechanistic similarity of the oxidative addition step and that this step initiates both catalytic cycles (Scheme 9C). Subjecting an equimolar mixture of 4-phenyltetrahydropyran **37** and 4-chlorotetrahydropyran **30** to Grignard reagent in the presence of nickel catalyst resulted in formation of a 1:1.2 mixture of Kumada-type and cyclopropane products. This result is consistent with the proposal that both catalytic cycles are initiated by virtually identical oxidative addition events.

For XEC reactions of 1,3-dielectrophiles, we have determined that the new C–C bond of the cyclopropane is formed via a stereospecific intramolecular S_N_2-type reaction. Importantly, this mechanism does not correlate to those commonly reported for other XEC reactions, including the sequential reduction and radical chain mechanisms proposed for intermolecular XEC reactions.^26^ Sequential reduction and radical chain mechanisms typically involve two net OA events (via polar or radical intermediates) and four oxidation states of the catalyst [Ni(0), Ni(I), Ni(II), and Ni(III) intermediates], and the C–C bond of the product is generated by reductive elimination.^27^ Our mechanism involves only one OA event and only two catalyst oxidation states [Ni(0) and Ni(II) intermediates].

**Scheme 10:**
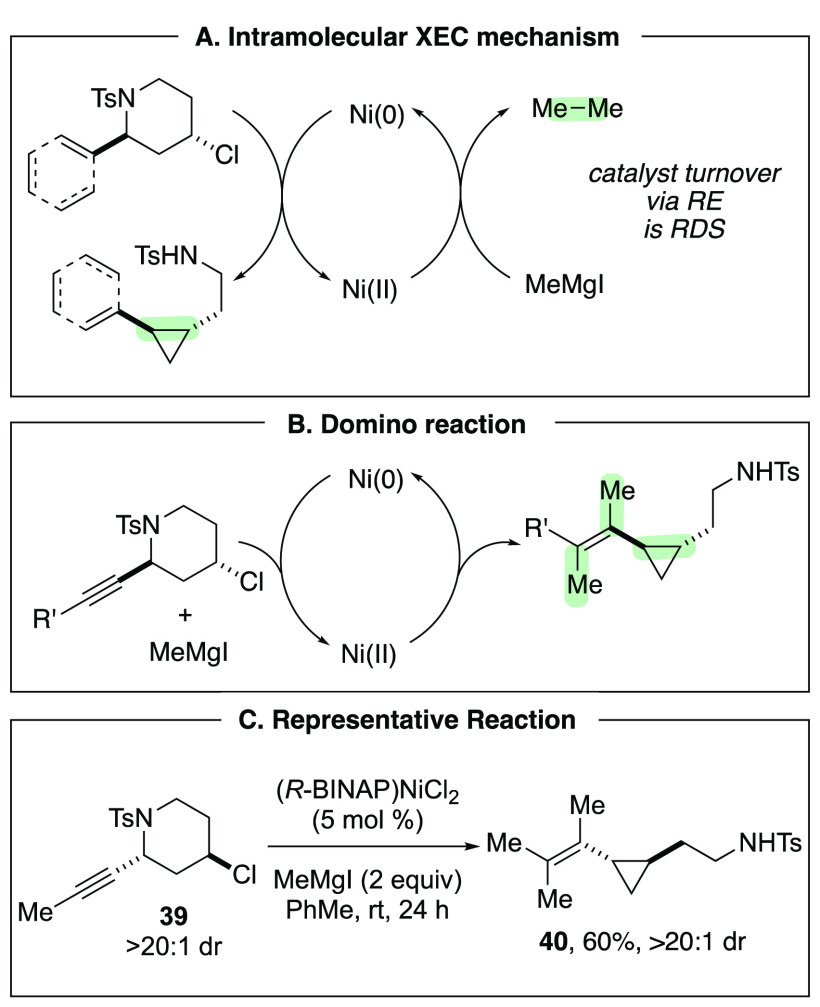
Domino Reaction of Alkynylpiperidines

We hypothesized, based on our mechanistic evidence, that the resting state for intramolecular XEC reaction is a nickel(II) intermediate,^28^ providing an opportunity to further engage the catalyst in additional C–C bond forming steps (Scheme 10A). We have established a domino reaction of 2-alkynylpiperidines, which accomplishes ring contraction of the 4-chloropiperidine moiety as well as dicarbofunctionalization of the alkyne moiety (Scheme 10B,C).^29^ This reaction is similar to our other XEC reactions, as we hypothesize that the first step is oxidative addition of the activated C–N bond.

### Part II: Stereoablative Reactions of Alcohol Derivatives

**Scheme 11:**
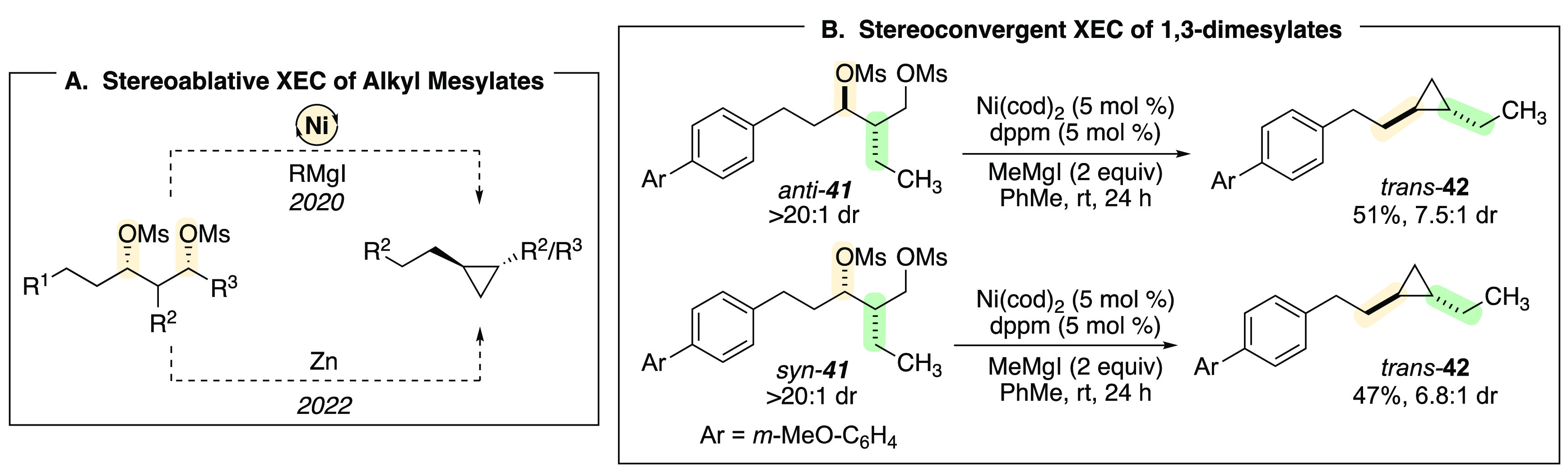
Stereoconvergent XEC Reactions of 1,3-Dimesylates

In order to engage a broader range of substrates, we sought alternative methods for activation of alkyl alcohols. For benzylic and allylic ethers, ligation by the arene or olefin facilitates the oxidative addition step, and a π-benzylnickel intermediate is generated, such that a poor leaving group (typically alkoxide) is tolerated. To approach less activated alkyl substrates, where ligation is not possible and subsequent organonickel complexes are not stabilized by conjugation, we examined a series of activating groups. Our group first demonstrated this concept in an intramolecular XEC reaction of 1,3-dimesylates (Scheme 11A). Under similar reaction conditions to those employed with benzylic ethers, in the presence of a nickel catalyst and stoichiometric Grignard reagent, 1,3-dimesylates such as **41** are converted to cyclopropanes. Notably, the reaction is stereoablative with respect to the secondary mesylate center, with both diastereomers of dimesylate **41** providing *trans*-cyclopropane **42** as the major product (Scheme 11B).

**Scheme 12:**
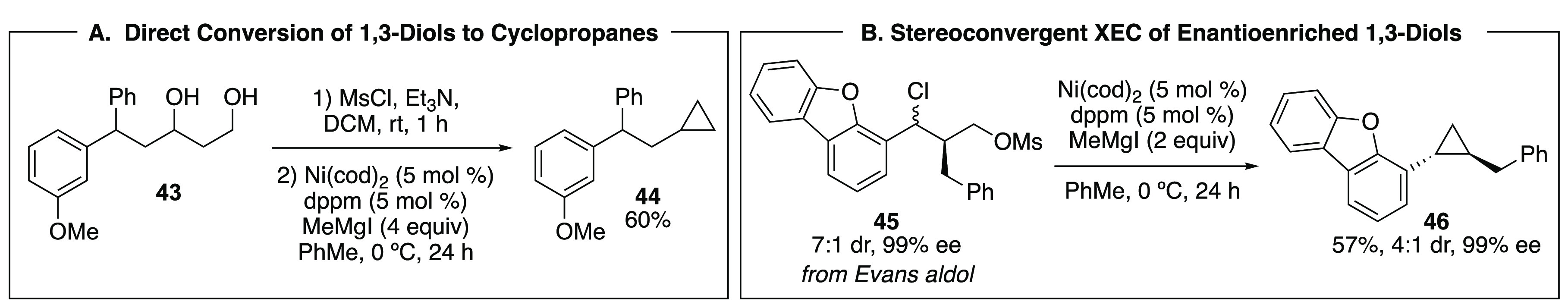
In Situ Conversion of Readily Accessible 1,3-Diols to Cyclopropanes

We developed the scope of this reaction to include a broad range of alkyl 1,3-diols, including establishing one-pot conditions for direct conversion of 1,3-diols to cyclopropanes (Scheme 12A). A significant benefit of this reaction is that starting materials can be prepared by enantioselective aldol reactions, providing straightforward access to enantioenriched cyclopropanes (Scheme 12B). For example, an Evans aldol reaction was employed to prepare enantioenriched mesylate **45**. Subsequent nickel-catalyzed XEC reaction proceeded to provide the desired *trans*-cyclopropane **46** in 99% ee.

**Scheme 13:**
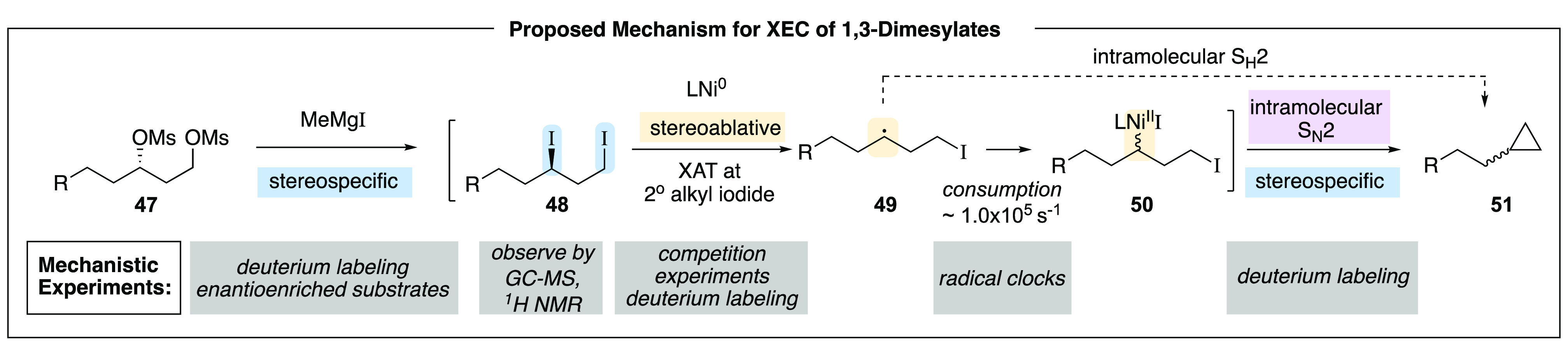
Mechanistic Investigation of XEC of 1,3-Dimesylates

To better understand and further develop this reactivity manifold, we have examined the mechanism of the reaction.^30^ We determined that, in addition to serving as the terminal reductant, the Grignard reagent serves as a source of nucleophilic iodide and transforms dimesylate **47** to diiodide **48** in situ in a stereospecific S_N_2 step.^31^ Subsequent XAT of the secondary alkyl iodide generates an alkyl radical (**49**) that rapidly epimerizes.^32,33^ Based on radical clock experiments, this radical is consumed at a rate competitive to 5-exo-trig cyclization. Secondary radical **49** can be captured to generate organonickel(II) complex **50**. Based on analysis with deuterium-labeled substrates, subsequent stereospecific ring closure provides cyclopropane **51**, proceeding with inversion at the primary electrophilic center (Scheme 13). Computational results are consistent with formation of organonickel complex **50**; however, direct S_H_2 cyclization of secondary radical **49** is also possible. Since both pathways are expected to be stereospecific and proceed with inversion at the electrophilic carbon, we have not yet been able to distinguish between these two mechanisms experimentally.

**Scheme 14:**
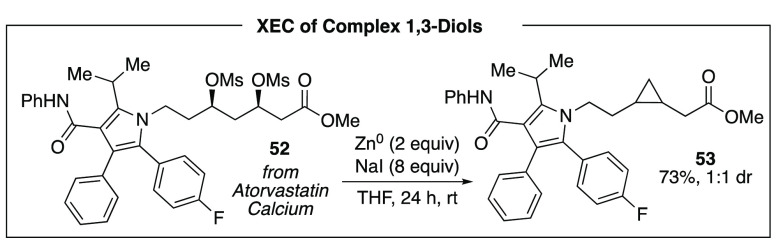
Zn-Mediated Synthesis of Cyclopropanes from Complex 1,3-Diols

In order to improve the functional group compatibility of the XEC reaction, we sought to replace the unusual reducing agent, MeMgI, with a metal powder such as Zn or Mn, which are more commonly employed in related XEC reactions.^24^ After evaluation of a series of reaction conditions, we determined that zinc powder, in the absence of a nickel catalyst, was capable of transforming dimesylates to cyclopropanes (Scheme 14).^34^ Critical to the reaction was addition of stoichiometric quantities of an iodide salt, NaI, consistent with formation of alkyl iodides in situ as key intermediates. With these reaction conditions, we can engage a range of substrates bearing sensitive functional groups including a series derived from statins.

**Scheme 15:**
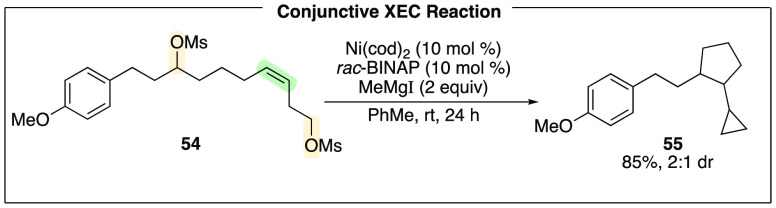
Synthesis of Vicinal Carbocycles from Alkyl Dimesylates

With an understanding of the key steps of the mechanism that allow for activation of the alkyl mesylate and formation of an alkyl radical, we also sought to employ this reactivity manifold for complex ring formation. A series of dimesylates, separated by pendant olefins, underwent smooth cascade reactions to afford vicinal carbocycles such as **55** (Scheme 15).^35^ These reactions proceed with modest diastereomeric ratios consistent with radical intermediates.

**Scheme 16:**
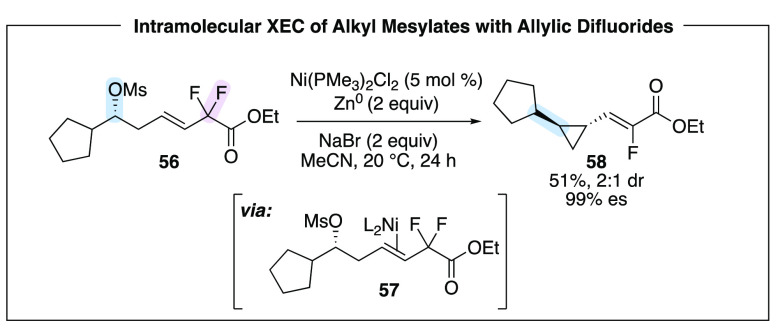
Allylic Difluorides as Intramolecular Coupling Partners

To further the scope and reactivity parameters of intramolecular XEC reactions, we sought to pair an alkyl mesylate with a different electrophile, as we had accomplished in the stereospecific series (e.g., Scheme 6). We hypothesized that allylic difluorides could serve as a suitable coupling partners. Upon subjection to the reaction conditions, we were pleased to see that mesylate **56** underwent nickel-catalyzed transformation to the desired fluorinated vinylcyclopropane **58** (Scheme 16).^36^ During reaction optimization, we recognized that this transformation was significantly different than the XEC of 1,3-dimesylates. For example, in the presence of zinc as the reducing agent, the nickel catalyst was still required. In addition, iodide salts were detrimental to the reaction and bromide salts provided higher yields and fewer byproducts. Furthermore, we were surprised to observe that the reaction was enantiospecific and occurred cleanly with inversion at the alkyl mesylate. This result rules out a mechanism which involves formation of an alkyl radical intermediate. Experimental mechanistic investigations and theoretical computations performed by the Hirschi laboratory were consistent with formation of a stable olefin complex (**57**) that precedes oxidative addition. Indeed, low-valent nickel catalysts are known to have a strong affinity for electron-poor alkenes.^37^ These results underscore the continued mechanistic ambiguity associated with development of new nickel-catalyzed reactions.

### Part III: Multivariable Control of Mechanism of Oxidative Addition

One challenge for the future of the field of nickel-catalyzed coupling reactions is reliable and predictive understanding of features that control the reactivity of nickel catalysts. Future reaction development is more fruitful when it can be guided by straightforward principles that are predictive toward reaction yield and stereochemical outcome. Indeed, mechanistic analysis in the context of palladium coupling reactions provided guidelines for ligand selection that propelled the field and contributed to a wide range of researchers embracing and employing XC reactions.^38^ When compared to palladium catalysts, the possibilities for ligand-based mechanistic control are even greater with nickel catalysts due to their propensity to participate in a broader range of elementary steps. Therefore, delineating design principles that can favor certain pathways could be even more impactful.

A design feature that was of interest to us was the control of the mechanism of oxidative addition for C(sp^3^)-hybridized electrophilic carbons. Oxidative addition may occur by a two-electron reaction that mirrors a traditional S_N_2 reaction and occurs with inversion. Alternatively, oxidative addition may proceed via one-electron steps via formation of an alkyl radical. The one-electron pathways are stereoablative, since the alkyl radical racemizes (or epimerizes) rapidly.^30,39^ For transformations that construct new C(sp^3^) stereogenic centers, favoring one mechanism for oxidative addition is critical in controlling the overall stereochemical outcome of the reaction.

**Figure 4:**
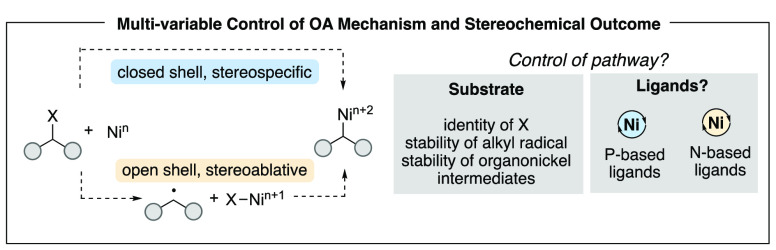
Multivariable control in nickel-catalyzed reactions.

On the basis of a broad range of XC and XEC reactions, we hypothesized that two variables control the mechanism of oxidative addition with alkyl electrophiles (Figure 4). The first, and most obvious, is the structure of the substrate, and in particular, the identity of the leaving group. The second feature appeared less obvious. On the basis of our analysis of literature examples as well as unpublished work from our own laboratory, we hypothesized that the ligand also played a role in determining whether oxidative addition proceeded smoothly and by which mechanism. In particular, we hypothesized that phosphine-based ligands tend to provide facile S_N_2-type oxidative addition, while nitrogen-based (pyridyl-, oxazoline-, imidazoline-, and amine-type) ligands tend to facilitate one-electron reactions that generate alkyl radicals (Figure 4). It is important to note that while the oxidation state of the catalyst is thought to differ between these modes of oxidative addition–nickel(0) complexes are thought to undergo two-electron oxidative addition while nickel(I) complexes are more prone to one-electron reactions; choice of precatalyst cannot reliably control the oxidation state of the active catalyst. Rapid redox reactions occur in situ, such that the oxidation state of the active complex may not match that of the precatalyst.^40−42^ We have demonstrated that for certain reactions, nickel(0), nickel(I), and nickel(II) precatalysts all rapidly generate nickel(0) in situ and are indistinguishable from a kinetic perspective.^26^ Therefore, the oxidation state of the precatalyst will not provide predictive control over the oxidative addition mechanism. However, we hypothesized that the identity of the ligand could indeed favor intermediates of certain oxidation states, as well as influence the barrier heights for key elementary steps.

**Scheme 17:**
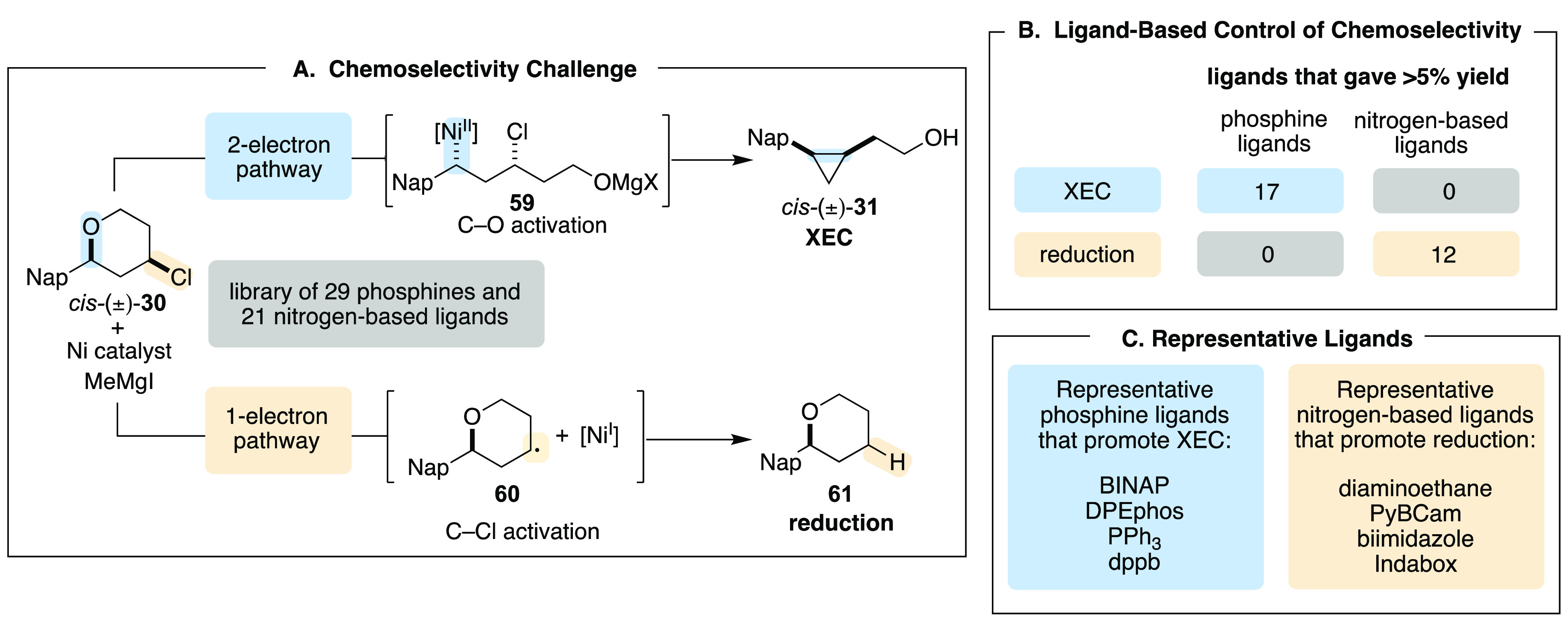
Probing Ligand-Based Control of Chemoselectivity

We sought a control reaction to test our hypothesis, where the catalyst would have the opportunity to engage in one- or two-electron pathways with a single substrate under a single set of reaction conditions. Divergent reactions of tetrahydropyran **30** provided this test reaction (Scheme 17A).^43^ This substrate presents two electrophilic functional groups, a benzylic ether and an alkyl chloride. Alternatively, oxidative addition of the benzylic ether, which proceeds via a two-electron, S_N_2-like transition state, leads to formation of cyclopropane **31** as the product. Oxidative addition of the alkyl chloride was established to proceed by XAT, and formation of the alkyl radical **60** leads to formation of reduced product **61**. Therefore, subjecting tetrahydropyran **30** to standard reaction conditions and varying the ligand would interrogate the ability of ligands to promote one reaction manifold over the other.

We examined a series of 50 ligands, including many that are commonly employed in XC and XEC reactions. A selection of monodentate and bidentate phosphines, as well as nitrogen-based ligands including bipy derivatives and diamines, were incorporated. Interestingly, across the 50 ligands, only phosphine ligands provided >5% yield of the cyclopropane XEC product **31**, resulting from two-electron oxidative addition (Scheme 17B,C). In contrast, significant amounts of reduced tetrahydropyran **61** were only observed when nitrogen-based ligands were employed. These results are consistent with ligand-based control of electrophile preference and mechanism of oxidative addition. Further studies are ongoing to determine the generality of this trend and uncover the rationale that underlies the divergent reactivity preferences.

## Conclusions and Future Directions

In this Account we have highlighted our laboratory’s contributions to the field of nickel-catalyzed XC and XEC reactions. We have demonstrated activation of benzylic C–O bonds in stereospecific Kumada-, Suzuki-, and Negishi-type C(sp^3^)–C(sp^3^) XC reactions and adapted those principles to the development of new XEC reactions. By activation of alkyl alcohols as the corresponding mesylates, we established stereoconvergent intramolecular XEC reactions of 1,3-diols. The methods developed and mechanistic analysis of the reaction pathways increase the potential for application of alkyl alcohol derivatives as electrophiles for XC and XEC reactions. Finally, we have begun to investigate the reaction features which differentiate stereospecific from stereoablative oxidative addition pathways and established that there is an interplay between multiple variables including leaving group and ligand structures. Specifically, comparison of phosphine-based versus nitrogen-based ligands demonstrates that they play a distinct role in facilitating formation of closed- versus open-shell intermediates. Future challenges for the field include continued development of new C(sp^3^)–C(sp^3^) bond-forming reactions for late-stage functionalization of alcohols. For example, intramolecular reactions that generate a range of ring sizes (e.g., from 1,4-diols, 1,5-diols, and 1,6-diols) would provide exciting annulation strategies that would have profound impact on the conformations of complex diols. Expansion to include intermolecular, cross-selective reactions of two diols would also be an important step forward for functionalization (e.g., methylation) as well as fragment coupling. From a mechanistic perspective, further definition of the key features of multivariable control in nickel-catalyzed XC and XEC reactions will be critical in improving our understanding of the factors that underlie reactivity and our ability to predict and design new selective catalytic transformations.
